# Uses of the Nitro-Muriatic Acid Bath

**Published:** 1851-03

**Authors:** Ranald Martin


*Uses of the Nitro-Muriatic Acid Bath.* By Ranald Martin, Esq.—Mr. Martin, in some valuable papers on tropical diseases, gives the following directions for the preparation and use of the acid bath:

Take of hydrochloric acid, three parts; nitric acid, two parts; distilled water, five parts, mix carefully.

1. Of this dilute nitro-muriatic acid, three ounces by measure are to be added to each gallon of water, to form the bath.

2. Two gallons of water may suffice for an ordinary foot-bath.

3. The bath thus prepared will keep in use for a week, by adding to it, once every day, half an ounce of the dilute acid, and a pint of water, in order to make up for waste in evaporation.

4. A portion only of the bath is to be heated for use, after which it is to be added to the remainder, so as to make the whole of from 96° to 98°.

5. Let both feet be placed in the bath, while the inside of the legs and thighs, the right side over the liver, and the inside of both arms, are sponged alternately; or let the abdomen be swathed in flannel soaked in the bath. This should be continued for fifteen minutes, morning and evening.

6. While using the bath, a gentle aperient, as Cheltenham or Epsom salts, in some bitter infusion, should be taken every other morning; and, should there be dryness or harshness of the skin, a warm vapor-bath, used twice a week, will be found of much service in stimulating and opening the pores, and in purifying the surface of the body.

7. In urgent cases, a general bath to envelope the whole body should be used, the proportions of the dilute acid and water being continued as above stated.

8. Earthen or wooden vessels should be used for the baths, and the sponges and towels kept in cold water, lest the acid corrode them.

9. The acid mixture forming the bath should be heated in earthenware vessels—large pipkins for instance. When used, the temperature should be measured by the thermometer, at 96° and 98°, as the body will be chilled by a degree of warmth that feels comfortable to the hand.

10. When a general bath is used for the whole body, the patient, before going into the bath, should be covered over with blankets, until a gentle perspiration is induced.

11. When in the bath, a covering blanket should be drawn over the shoulders, and up to the chin, to confine the steam, and a night-cap should be worn to protect the head from the steam.

12. Before quitting the bath, the bed, drawers, and towels should be warmed and ready; the body to be dried while standing in the bath, and the dressing to be performed immediately.

13. When the acid-bath excites much irritation of the skin, the quantity of dilute nitro-muriatic acid may be diminished; and when irritation of the gums, with general malaise, occurs, the use of the bath should be relinquished for a time, resuming its application, if necessary, when the above symptoms have subsided.

Mr. Martin states that a course of two months of this bath is found generally to restore the healthy action of the liver; nor does it interfere with the use of bitter tonics, or even with mild chalybeates, but, on the contrary, it rather gives effect to their operations. In the severer instances he has continually to use this means for a much longer period. In treating torpor of the liver, he recommends warm and tepid baths, and especially those of sea-water, in aid of other medical treatment, reducing the temperature as the patient approaches health, and after the cessation of other means of cure.

The neglect of the nitro-muriatic acid treatment in cases of the nature here treated of, and in which mercury in every form, proves not only insufficient but pernicious, is much to be regretted.

A valuable quality of the nitro-muriatic acid is, that whether exhibited internally or externally, its influence in promoting the secretions is not in the least degree counteracted by opium, even when exhibited in the largest doses. We obtain, in short, from the use of the mineral acids in the treatment of chronic disease, all the remedial aids which can be obtained from mercury in the treatment of acute disease; and that, without any of the injurious consequences that so frequently ensue from the exhibition of the last named agent. Persons of anemic or of scrofulous habit are liable to serious injury from the use of mercury, whether given in large doses, during a few days, or in alterative doses during weeks or months. It is quite otherwise in respect to the acids, the most marked and lasting improvement of the general health being the ordinary result of their exhibition.—*Lancet and Prov. Med. and Surg. Jour.—Ib.*

